# Commentary: A posterior-to-anterior shift of brain functional dynamics in aging

**DOI:** 10.3389/fnagi.2019.00341

**Published:** 2019-12-10

**Authors:** Ping Ren, Mia Anthony, Dag Aarsland, Donghui Wu

**Affiliations:** ^1^Shenzhen Mental Health Center, Shenzhen, China; ^2^Department of Geriatric Psychiatry, Shenzhen Kangning, Shenzhen, China; ^3^School of Nursing, University of Rochester Medical Center, Rochester, NY, United States; ^4^Department of Old Age Psychiatry, Institute of Psychiatry, Psychology and Neuroscience, King's College London, London, United Kingdom

**Keywords:** aging, posterior-to-anterior shift, resting-state fMRI, compensation effect, functional dynamics

In the late stages of life, the brain undergoes remarkable structural and functional changes, which lead to cognitive decline. Evidence from functional magnetic resonance imaging (fMRI) studies has shown widespread changes of regional activity and functional connectivity in aging brain, including the prefrontal cortex (PFC), temporal lobe (TL), and subcortical structures (Damoiseaux et al., 2008; Ferreira and Busatto, 2013). To explain cognitive aging by incorporating neuroimaging evidence, the Scaffolding Theory of Aging and Cognition (STAC) posits that the brain alterations in aging may underlie a compensatory process of cognitive scaffolding, in which cognitive functions could be protected via recruitment of additional brain regions and/or networks (Goh and Park, 2009; Park and Reuter-Lorenz, 2009; Sala-Llonch et al., 2015). In accordance with the STAC, a posterior-to-anterior shift in aging (PASA) has been found in different kinds of cognitive tasks using task-related fMRI, showing an age-related decreased activation in occipitotemporal regions coupled with increased activation in frontal regions (St Jacques et al., 2010; Ansado et al., 2012). Resting-state fMRI, which measures regional interactions in the absence of an external task, has been widely used in scientific and clinical research due to its simplicity and reliability. Although the PASA phenomenon has been documented in a variety of task-related fMRI studies, the neural basis that underlie the cerebral reorganization in aging remains poorly understood. Moreover, there has been less evidence in support of PASA in resting-state brain fMRI studies.

Zhang and his colleagues investigated the PASA hypothesis by examining brain functional dynamics of resting-state blood-oxygen-level-dependent (BOLD) signals, in a large sample (*n* = 277) aged from 22 to 79 (Zhang et al., 2017). Brain dynamics during rest has been found associated with processing efficiency and cognitive demand (Cole et al., 2016). In their study, parcellations from the Automated Anatomical Labeling (AAL) atlas were grouped into 12 large regions as regions of interest (ROIs) for extracting the brain functional dynamics. Functional hub probability was calculated to characterize functional hub dynamics, with lower hub probability indicating lower stability to be a hub. Subsequently, dynamic functional connectivity was computed to measure the temporal variability of functional connectivity between each pair of the ROIs. According to the PASA model, aging brain shows hypoactivity in the posterior regions and hyperactivity in the anterior regions in response to cognitive decline. Thus, authors postulated that the frontal regions would become less stable to be hubs as age increased, accompanied by recruiting additional regions as hubs in the occipital regions. This could be reflected by: (1) a decreased hub probability in the anterior brain and an increased hub probability in the posterior brain; (2) an enhanced variability of the functional connectivity in the frontal lobe to adapt to cognitive challenges in aging.

The results showed significant negative effects of age on functional hub probability in the anterior brain regions, including the PFC, sensorimotor cortex (SMC), and subcortical structures. In contrast, they found significant positive effects of age on functional hub probability in the posterior brain regions, including the temporal lobe (TL) and occipital cortex (OC). The decreased hub probability in the anterior brain suggest a more flexible role of frontal regions to adapt to cognitive needs in aging. In dynamic functional connectivity analysis, older adults showed augmented dynamics in the anterior regions, and attenuated dynamics mainly within the posterior regions. In other words, the frontal brain became more flexible in modulating its functional networks as reflected by increasing variability of its functional connectivity in aging. The consistent findings in functional hub probability and dynamic functional connectivity further support the PASA model in resting-state BOLD activities in older adults. Additionally, they found decreased dynamic functional connectivity of posterior regions associated with age-related episodic memory decline, which accounted for the disrupted posterior function in PASA.

Zhang's study provides new evidence on the hub probability and variability of functional connectivity in support of the STAC model in aging brain. More importantly, their findings demonstrate that the alterations of resting-state functional dynamics in aging brain is in line with the PASA hypothesis. The augmented recruitment of frontal regions in older adults may be a compensatory effect in response to aging-related cognitive impairment. Similarly, additional usage of frontal networks has been implicated as a compensatory response in multiple psychiatric disorders, including Alzheimer's disease (Grady et al., 2003), Parkinson's disease (Gerrits et al., 2015), and Huntington's disease (Georgiou-Karistianis et al., 2007). While these above findings are interesting, there are some limitations in Zhang and colleagues' study. First, they only involved a global screening instrument [the Mini-Mental State Examination (MMSE)] to assess the cognitive function in older adults, which was not sufficiently sensitive for identifying cognitive decline in aging. Thus, more specific neuropsychological assessments and cognitive tests should be involved in the future studies, such as the Montreal Cognitive Assessment (MOCA), Mini-Cog test and so on (Tsoi et al., 2015). Second, it is necessary to do more work to confirm the compensatory effect of frontal regions based on their findings, since there was no correlation between altered frontal dynamics and MMSE. In addition to functional dynamics, more indices of resting-state brain activity [e.g., amplitude of low-frequency fluctuations (ALFF)] should be involved to examine the “posterior-to anterior shift” in future studies. Third, Zhang and colleagues grouped the ROIs in AAL atlas to obtain 12 large regions, which needs to be validated using other functional or anatomical atlas.

Consistent findings from aging studies suggest that the frontal cortex is closely associated with compensatory effect in aging-related cognitive decline (Franzmeier et al., 2017b, 2018; Gonzalez-Escamilla et al., 2018). Based on previous studies and Zhang's findings, we argue that the “posterior-to anterior shift” may be a common mechanism of cerebral reorganization in response to cognitive impairment not just in normal aging, but also in psychiatric disorders. For instance, converging evidence has shown hyperactivation of frontal regions could be used to compensate cognitive deficits in Autism Spectrum Disorder (Livingston and Happe, 2017). Similarly, the compensatory process by recruiting additional fontal regions has been found in major depression (Chechko et al., 2013), schizophrenia (Sapara et al., 2014), Parkinson disease (Simioni et al., 2017), and Alzheimer's disease (Franzmeier et al., 2017a). Our recent work reported a similar posterior-to-anterior shift pattern in cognitive fatigue (CF) modulation in healthy older adults, suggesting an aging-associated neural reliance on frontal regions to compensate CF-induced cognitive inefficiency (Ren et al., 2019). In addition, the findings in cognitive reserve studies indicate that the brain functional and structural measurements for compensation could be influenced by combination of molecular and genetic factors (Bartres-Faz et al., 2019; Pietzuch et al., 2019). Thus, future studies need to explore the compensatory mechanism in the “posterior-to anterior shift” brain alteration, and reveal the differences between normal aging and psychiatric symptoms. More interestingly, the anterior and posterior brain regions are considered to play different roles in perceived consciousness according to two opposite theories: the global workspace theory (GWT) and integrated information theory (IIT) (Mashour, 2018). Recently, the Templeton World Charity Foundation (TWCF) funded a new project to explore the neural mechanism of consciousness by examining the GWT and IIT (Reardon, 2019). We speculate the “posterior-to-anterior shift” may play a critical role in altered states of consciousness in some psychiatric disorders (e.g., schizophrenia), which needs to be examined in future studies.

## Author Contributions

PR conceived the idea for this commentary and wrote the first draft. MA and DA revised drafts. DW improved the manuscript and added sections and references.

### Conflict of Interest

The authors declare that the research was conducted in the absence of any commercial or financial relationships that could be construed as a potential conflict of interest.
